# Activation of adenosine receptor A_2A_ increases HSC proliferation and inhibits death and senescence by down-regulation of p53 and Rb

**DOI:** 10.3389/fphar.2014.00069

**Published:** 2014-04-10

**Authors:** Md. Kaimul Ahsan, Wajahat Z. Mehal

**Affiliations:** Department of Internal Medicine, Section of Digestive Diseases, Yale UniversityNew Haven, CT, USA

**Keywords:** liver fibrosis, hepatic stellate cells, adenosine receptors, apoptosis, senescence

## Abstract

**Background and Aims:** During fibrosis hepatic stellate cells (HSC) undergo activation, proliferation, and senescence but the regulation of these important processes is poorly understood. The adenosine A_2A_ receptor (A_2A_) is known to be present on HSC, and its activation results in liver fibrosis. In this study, we tested if A_2A_ has a role in the regulation of HSC proliferation, apoptosis, senescence, and the relevant molecular mechanism.

**Methods**: The ability of adenosine to regulate p53 and Rb protein levels, proliferation, apoptosis and senescence was tested in the human HSC cell line LX-2 and rat primary HSC.

**Results**: Adenosine receptor activation down-regulates p53 and Rb protein levels, increases BrdU incorporation and increases cell survival in LX-2 cells and in primary rat HSC. These effects of NECA were reproduced by an adenosine A_2A_ receptor specific agonist (CGS21680) and blocked by a specific antagonist (ZM241385). By day twenty-one of culture primary rat HSC entered senescence and expressed β-gal which was significantly inhibited by NECA. Furthermore, NECA induced down regulation of p53 and Rb and Rac1, and decreased phosphorylation of p44-42 MAP Kinase in LX-2 cells and primary rat HSC. These effects were reproduced by the cAMP analog 8-Bromo-cAMP, and the adenylyl cyclase activator forskolin, and were blocked by PKA inhibitors.

**Conclusions**: These results demonstrate that A_2A_ receptor regulates a number of HSC fate decisions and induces greater HSC proliferation, reduces apoptosis and senescence by decreasing p53 and Rb through cAMP-PKA/Rac1/p38 MAPK pathway. This provides a mechanism for adenosine induced HSC regulation and liver fibrosis.

## Introduction

Activation of hepatic stellate cells (HSC) is recognized as a vital step in liver fibrosis and results in the acquisition of multiple functions including contraction, chemotaxis and matrix remodeling (Kawada et al., 1992; Rockey, 1995; Watanabe et al., 2007; Soon and Yee, 2008; Mohamadnejad et al., 2010). During fibrosis the activated HSC population is simultaneously undergoing proliferation, apoptosis and senescence (Houglum et al., 1997; Knittel et al., 1997; Iredale et al., 1998; Iredale, 2001; Krizhanovsky et al., 2008a). The induction of proliferation is an early step after HSC activation and is stimulated by a number of cytokines including platelet derived growth factor (PDGF) (Pinzani et al., 1989; Kinnman et al., 2001). HSC are also undergoing apoptosis during the progression of fibrosis and this increases during fibrosis resolution (Reinehr et al., 2008). Overall HSC are resistant to the usual initiators of apoptosis such as CD95-L and oxidiative stress, and *in vivo* HSC apoptosis is induced by natural killer cells and Kupffer (Fischer et al., 2002; Radaeva et al., 2006).

The central features of senescence is irreversible growth arrest, an enlarged cellular morphology and expression of senescence-associated beta-galactosidase (SA-Bgal) (Hayflick, 1965; Dimri et al., 1995; Campisi, 2011). Although cellular senescence was first described in 1965 in normal human cells in culture its occurrence *in vivo* was only confirmed recently when it was shown that the fibrotic liver contains a number of senescent cells, and these were identified to be predominantly HSC (Krizhanovsky et al., 2008b). Furthermore if HSC were genetically modified to stop the development of senescence, they continued to proliferate resulting in increased fibrosis. The development of senescence is known to be dependent on the p16-Rb and Arf-p53-p21 pathways, and the modulation of these pathways can regulate cellular senescence (Abriss et al., 2003; Campisi and d'Adda di Fagagna, 2007).

The decision of HSC fate between proliferation, apoptosis and senescence clearly has a very significant effect on the development of fibrosis, yet little is known about how the entry of HSC between these three different states is regulated. A central pro-fibrotic role of adenosine via the A_2_ receptor has been identified, and adenosine results in increased production of collagen and transforming growth factor-β (Chan et al., 2006; Che et al., 2007; Sohail et al., 2009). The *in vivo* significance of adenosine is shown by the fact that mice lacking the A_2A_ receptor have reduced liver fibrosis(Chan et al., 2006). Because of this we hypothesized that adenosine may be an important regulator of HSC fate. Adenosine is very well suited for regulating HSC fate decisions as it is produced rapidly in the local environment in response to cell stress and damage, and has a very short half-life (Feoktistov et al., 2009; Chan and Cronstein, 2010).

This study demonstrates that adenosine, via the A_2A_ receptor down-regulates p53, and Rb and enhances proliferation of HSC cell lines and primary cells. This is associated with a reduction in HSC apoptosis, and senescence by via a PKA/Rac1/P38 MAPK pathway. This places adenosine as a key regulator of these key HSC fate decisions which determine the degree of liver fibrosis.

## Materials and methods

### Reagents

Pan adenosine receptor agonist 5′-N-Ethylcarboxamide adenosine (NECA), an analog of cAMP 8-Bromoadenosine 3′:5′-cyclic monophosphate (8-Bromo-cAMP), an adenylyl cyclase activator forskolin, and a PKA inhibitor H-89 dihydrochloride hydrate Sigma-Aldrich (St. Louis, MO). Adenosine receptor A_2A_ receptor antagonist ZM241385 and agonist CGS21680 Tocris Bioscience (Minneapolis, MN). Myristoylated PKA inhibitor 14–22 amide was purchased from Calbiochem (La Jolla, CA).

### LX-2 cell culture

Human immortalized hepatic stellate cell line, LX-2 have been previously described (Xu et al., 2005). Cells were cultured in high glucose (4.5g/L D-Glucose) containing Dulbecco's modified Eagle medium (DMEM), 100 IU/ml penicillin, 100 mg/ml streptomycin and 5% fetal bovine serum (Invitrogen). Cells were maintained at 37°C in a humidified atmosphere with 5% CO_2_. Culture media were replaced every 3 days.

#### Isolation and culture of HSC

**Figure 1:**
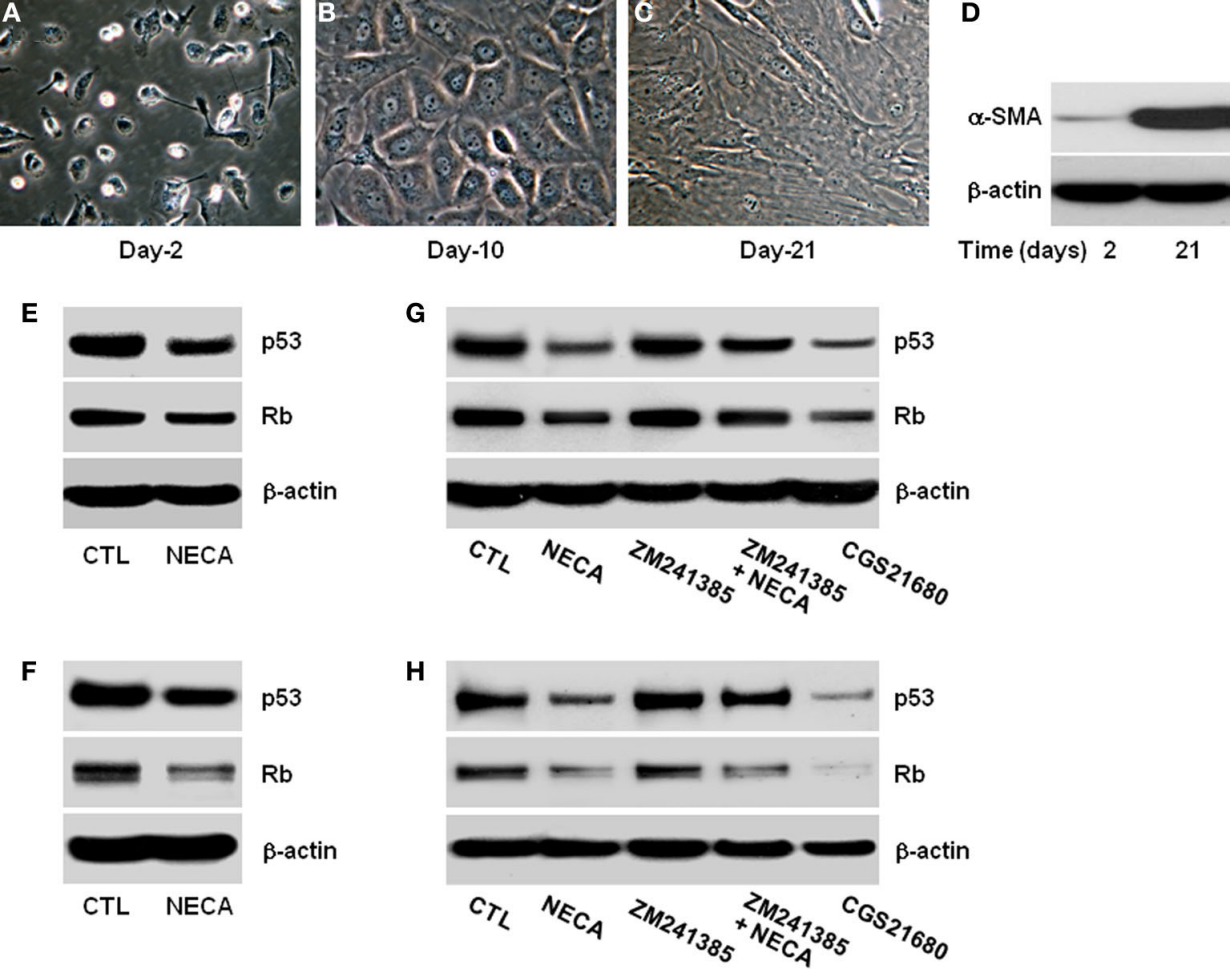
**Pan-adenosine receptor agonist (NECA) down regulates p53 and Rb level in HSC through adenosine receptor A_2A_ signaling**. Morphological characteristics of primary rat HSC were recorded for 2–21 days in the culture and shown **(A)** at day-2 in quiescent stage, **(B)** at day-10 in activated stage, or **(C)** at day-21 in highly activated stage. **(D)** Western blot shows expression of α-smooth muscle actin (α-SMA) at day-2 and at day-21 as a marker of activated primary HSC. Human HSC line LX-2 cells were treated for 2 days with or without 10 μM of NECA in presence or absence of either an A_2A_ receptor antagonist ZM241385 (10 μM) or agonist CGS21680 (10 μM). The control cells (CTL) were treated with DMSO. Primary rat HSC were similarly treated with NECA, ZM241385 and, or CGS 21680 for 5 days after 10 days normal culture (at the activated stage). **(E–G)** Western blots of p53 and Rb are shown for LX-2 **(F–H)** and primary rat HSC.

Male Sprague-Dawley rats (500–700 g) were used for primary HSC isolation. Experiments were performed in accordance with Institutional Animal Care and Use Committee. HSC were isolated via *in situ* pronase/collagenase perfusion followed by Opti-Prep density gradient centrifugation as described previously (Radaeva et al., 2007). HSC were plated in 10% FBS at a density of 1 × 10^5^ cells/ml and then plated onto 6-well plates in 2.5 ml cell suspension/well. After 12 h, non-adherent cells and debris were removed by washing with HBSS. Purity of the cultures was assessed by typical light microscopic appearance at this point. Vitamin A auto fluorescence was more than 90% (Figures 1A–D).

#### Western blots

Western blot analysis and immuno-detection was performed by standard protocols for 2–21 days cultured primary rat HSC or human HSC cell line LX-2 cells with indicated treatment. Blots were incubated with primary antibodies against α-smooth muscle actin (α-SMA; 1:2000; Sigma-Aldrich, St. Louis, MO), p53 and Rb (1: 1000; BD Phamingen, San Jose, CA), Rac1 (1: 1000; Millipore, Billerica, MA), phospho-p-44/42 MAPK and p-44/42 MAPK (1:1000; Cell Signaling Technology, Danvers, MA) or β-actin (1:1000; Santa Cruz Biotechnology) for overnight at 4°C followed by 1 h incubation at room temperature with HRP-linked IgG secondary antibodies. Blots were visualized by using a SuperSignal® West Pico chemiluminescent substrate (Thermo Scientific, Rockford, IL). β-actin was developed as a loading control. Cell proliferation was determined by 5-bromo-2′-deoxy-uridine (BrdU) incorporation assay using commercially available Cell Proliferation ELISA, BrdU (colorimetric) kit from Roche (Indianapolis, IN).

#### Annexin V/PI stained flow cytometric analysis of apoptosis

LX-2 cells were plated (2 × 10^5^ cells/well) in the 6-well plates and incubated overnight. Cells were treated with indicated reagents and cultured for 24 h. After the treatment cells were exposed to H_2_O_2_ (100 μM) for 5 h. Dead cells were quantified by annexin V-FITC-propidium iodide (PI) double staining, using Annexin V-FITC Apoptosis Detection Kit II (BD Biosciences). Experimental analysis was performed on FACS Calibur (BD, Arlington, MA) and the results were analyzed using WinMDI 2.9 Software (Joseph Trotter).

#### Senescence-associated β-galactosidase (SA-β-Gal) staining

Rat primary HSC were cultured in the 6 wells plate and SA-β-Gal staining was performed using a senescence detection kit (Calbiochem, La Jolla, CA). Cells were fixed in β-galactosidase fixative solution, washed with PBS and incubated overnight at 37°C with freshly prepared SA-β-Gal staining solution: 1 mg of 5-bromo-4-chloro-3-indolyl-β-D-galactoside (X-Gal)/ml (stock solution: 20 mg/ml in dimethylformamide) in staining solution with staining supplement. Thereafter cells were washed with PBS and β-galactosidase-positive cells were counted under bright field illumination using Image-J cell counting software.

#### Statistics

All data were shown as means ± s.e.m. Statistical significance were analyzed with unpaired student *t*-test using GraphPad Prism software (Version 5.0). *P* value is < 0.05 was taken as significant.

## Results

### Morphological changes of primary rat HSC during extended culture

HSC activation is characterized by gradual loss of retinoic acid and lipid stores, expansion of cytoplasm and gradual conversion to myofibroblasts leading to increase expression of α-smooth muscle actin (α-SMA) (Bataller et al., 2005; Friedman, 2008). We confirmed this in the *in vitro* culture of HSC on plastic. Day-2 after isolation HSC had the typical rounded and bright appearance (Figure 1A) and became gradually activated, with reduction in the retinoic acid and lipid droplets, expansion of cytoplasm by day-10 (Figure 1B), finally becoming large flatted myofibroblastic phenotype by day-21 (Figure 1C). These morphological changes were confirmed by assaying for the expression of α-SMA protein levels at day-2 and day-21. α-SMA was virtually absent at day-2, and had become highly upregulated by day-21 (Figure 1D).

### NECA suppresses p53 and Rb in LX2 and primary HSC through activation of adenosine receptor A_2A_

Since the expression of p53 and Rb levels are important initiator of senescence we analyzed their expression and protein level with or without treatment with the pan-adenosine receptor agonist NECA (Ben-Porath and Weinberg, 2005; Krizhanovsky et al., 2008b). NECA (10 μM) suppressed the expression of p53 and Rb protein levels in LX-2 (Figure 1E) and primary rat HSC (Figure 1F). Inhibition of p53 and Rb protein levels were reproduced by an A_2A_ receptor agonist (CGS21680 10 μM), and the ability of NECA to inhibit p53 and Rb protein levels were blocked by an A_2A_ receptor antagonist (ZM241385 10 μM) in LX-2 (Figure 1G) and primary rat HSC (Figure 1H). This demonstrates that p53 and Rb protein down-regulatory effects by NECA are through A_2A_receptor and its downstream signaling cascades. Furthermore, the slight upregulation of p53 and Rb protein levels in the presence of A_2A_ antagonist (Figures 1G,H) suggests that background levels of adenosine are functional in the usual tissue culture.

### NECA enhances cell proliferation through activation of adenosine receptor A_2A_ in the LX-2 cells and primary rat HSC

**Figure 2:**
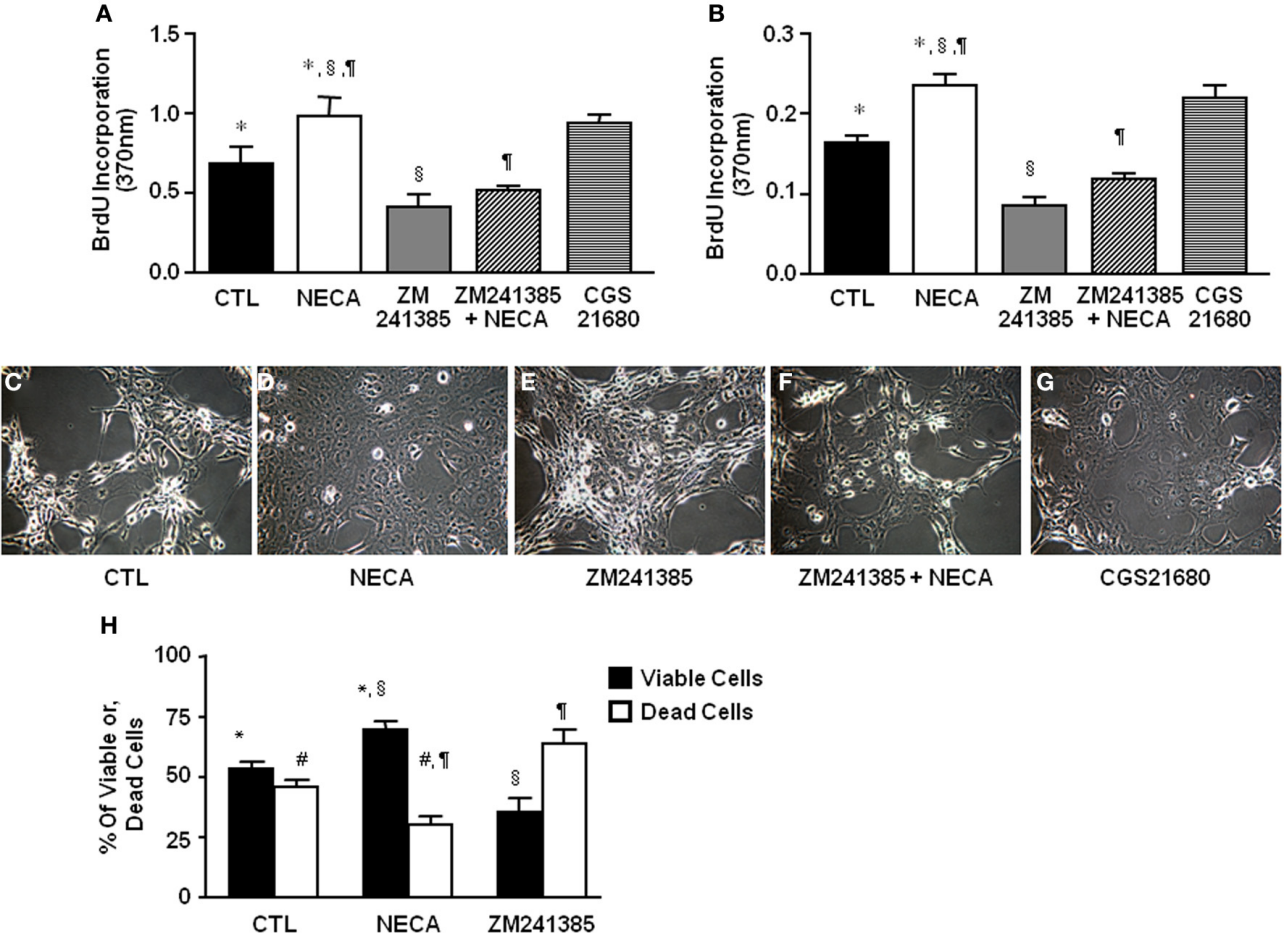
**NECA enhances BrdU incorporation and decreases H_2_O_2_-induced cell death in HSC through activation of adenosine receptor A_2A_**. LX-2 cells or 10 days *in-vitro* cultured primary rat HSC were treated with pan adenosine receptor agonist NECA (10 μM), an A_2A_ receptor antagonist (ZM241385 10 μM), or an A_2A_ receptor agonist (CGS21680 10 μM) for 24 h and measured BrdU incorporation. The level of BrdU incorporation was shown in **(A)** LX-2 cells (^*^ = 0.0126, ^§^ = 0.00099, ^¶^ = 0.00428) and in **(B)** primary rat HSC (^*^ = <0.001, ^§^ = <0.001, ^¶^= <0.001). LX-2 cells were treated **(C)** with vehicles DMSO, **(D)** NECA (10 μM), **(E)** an A_2A_ receptor antagonist (ZM241385 10 μM), or, **(F)** ZM241385 (10 μM) plus NECA (10 μM), or, **(G)** with an A_2A_ receptor agonist CGS21680 (10 μM) for 24 h followed by 100 μM of H_2_O_2_ for 5 h. **(H)** Viable and dead cells were quantified by both Annexin V and PI staining flow-cytometry (paired symbols mark comparisons with significance greater than *P* of 0.05). (^*^ = 0.0084, ^§^ = 0.0017, ^¶^ = 0.0017, ^#^ = 0.0084).

The ability of the pan-adenosine agonist NECA to downregulate p53 and Rb protein levels predicts that NECA will induce HSC proliferation. When tested NECA significantly increased cell proliferation by 0.971 ± 0.125 and 0.233 ± 0.015 over a 24 h period (*p* value = 0.001) in LX-2 (Figure 2A) and primary rat HSC (Figure 2B) respectively. This response was reproduced by an A_2A_ receptor specific agonist (0.93 ± 0.055 in LX-2 and 0.207 ± 0.006 in primary rat HSC), and completely blocked by an A_2A_ receptor antagonist in both LX-2 and primary rat HSC (Figures 2A,B).

### NECA inhibits H_2_O_2_-induced cell death in the LX-2 cells

HSC apoptosis is known to be a limiting mechanism for liver fibrosis. H_2_O_2_ provides a severe oxidative stress that induces HSC death. NECA-pretreated HSC were significantly resistant to H_2_O_2_ induced cell death. Data showed that 100 μM of H_2_O_2_ induced apoptosis by 5 h in control LX-2 (Figure 2C) which was inhibited by pretreatment with 10 μM of NECA (Figure 2D). A similar inhibition of H_2_O_2_-induced apoptosis was observed by the pretreatment with A_2A_ receptor agonist (10 μM) (Figure 2G). Pretreatment with an A_2A_ receptor antagonist significantly enhanced LX-2 cell apoptosis by 100 μM H_2_O_2_ compared to vehicle treated cells (Figure 2E). Furthermore, pretreatment with an A_2A_ receptor antagonist in addition to NECA significantly interfered with the ability of NECA-induced cellular survival against H_2_O_2_ (Figure 2F). Data were quantified by annexin V and propidium iodide (PI) staining FACS analysis in the Figure 2H. Long term culture of primary rat HSC (60 days) in the presence of NECA (10 μM) significantly enhanced cellular longevity compared to vehicle (DMSO)-treated cells (Supplemental Figure 1A), data were quantified in the Supplemental Figure 1B using Image-J cell count software. This demonstrates that NECA induced activation of A_2A_ receptor reduces HSC death in the presence of severe oxidative stress.

### NECA suppresses senescence and senescence-inducing markers p53 and Rb in the primary rat HSC

**Figure 3:**
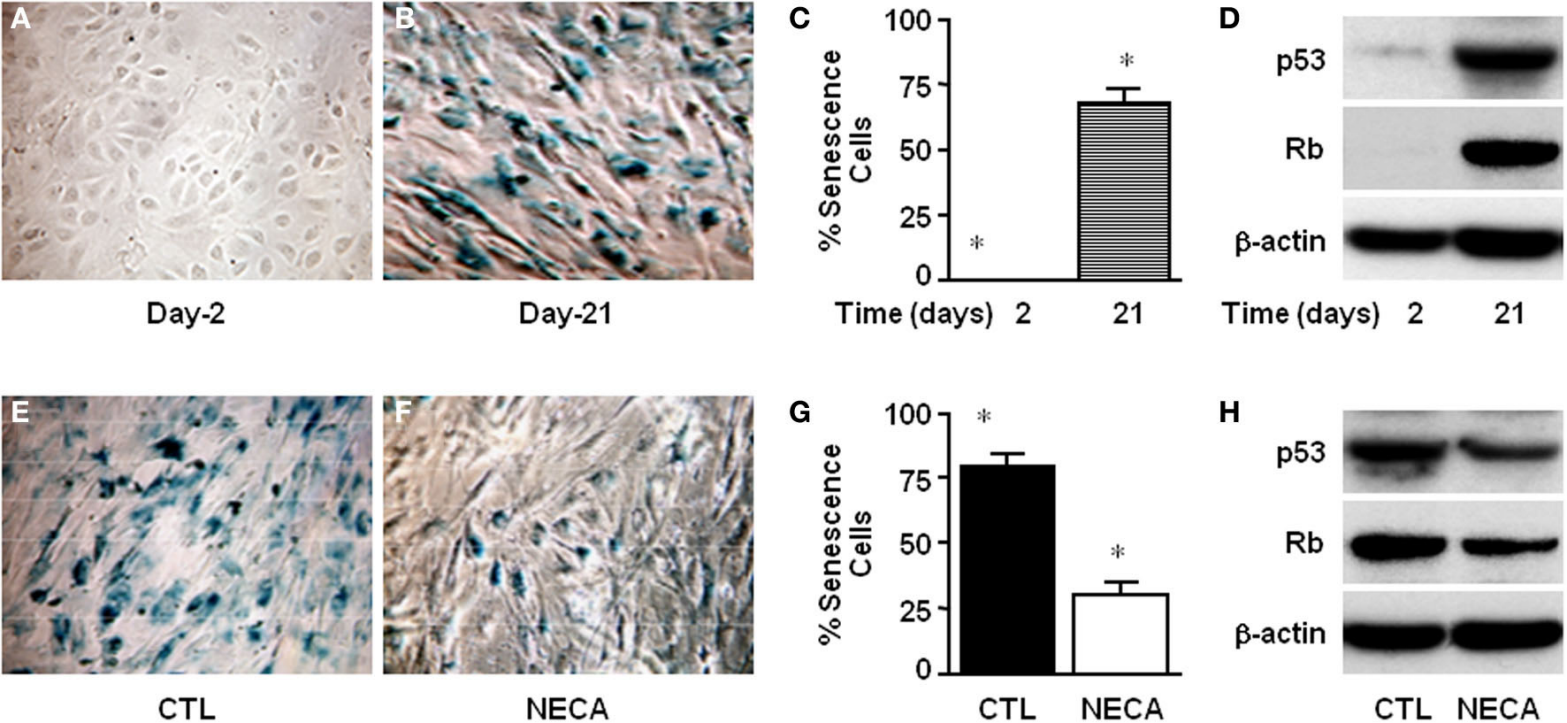
**NECA suppresses senescence by down regulating p53 and Rb in the primary rat HSC**. Cells were cultured for 2–21 days and senescence detected using SA-β-Gal staining **(A)** at day-2 or **(B)** at day-21, and **(C)** the number of senescent cells quantified (^*^ = significant but *p* value is not applicable because first column is totally zero). **(D)** Western blot analysis of p53 and Rb level in the normal culture at the day-2 or, day-21. Similarly senescence was detected in the HSC treated **(E)** with vehicle DMSO, or **(F)** with NECA for 21 days. **(G)** The number of senescence cells was quantified in the cells treated with DMSO (CTL) or, with NECA for 21 days using Image-J cell count software (^*^ = <0.001). **(H)** Western blot analysis of p53 and Rb level in the cells treated with DMSO (CTL) or, with NECA for 21 days.

Cellular senescence is a potent barrier to tumorigenesis and limits tumor cell proliferation and tumor evolution (Serrano et al., 1997; Schmitt et al., 2002; Braig et al., 2005; Krizhanovsky et al., 2008a). Moreover senescence of activated HSC limits proliferation thereby limiting liver fibrosis by down regulating production of extra cellular matrix (Krizhanovsky et al., 2008b). At day-2 of culture there was no SA-β-gal expression in primary rat HSC (Figure 3A). By day 21 of normal culture HSC had entered senescence (Figure 3B) up to 67.41 ± 6.11% (Figure 3C) as detected by SA-β-gal expression. This was supported by the very high level of expression of p53 and Rb at day-21 of culture compared with day-2 (Figure 3D). Treatment of HSC with 10 μM NECA for 21 days significantly reduced the degree of senescence to 29.60 ± 5.6% (Figures 3F,G) from 79.2 ± 4.9%, in control cells (Figures 3E–G; *p* value ≤ 0.001). NECA resulted in down regulation of both p53 and Rb (Figure 3H) at day 21 of culture. For the LX-2 cell line after 40 passages senescent LX-2 cells were detected with or without DMSO (Supplemental Figures 2A,B), however we were unable to detect any senescent LX-2 cells in either passages-2 (Supplemental Figure 2A) or NECA treated passages-40 (Supplemental Figure 2B).

### NECA suppresses p53 and Rb through PKA/Rac1/p38MAPK signaling cascades in the LX-2 cells and primary rat HSC

**Figure 4:**
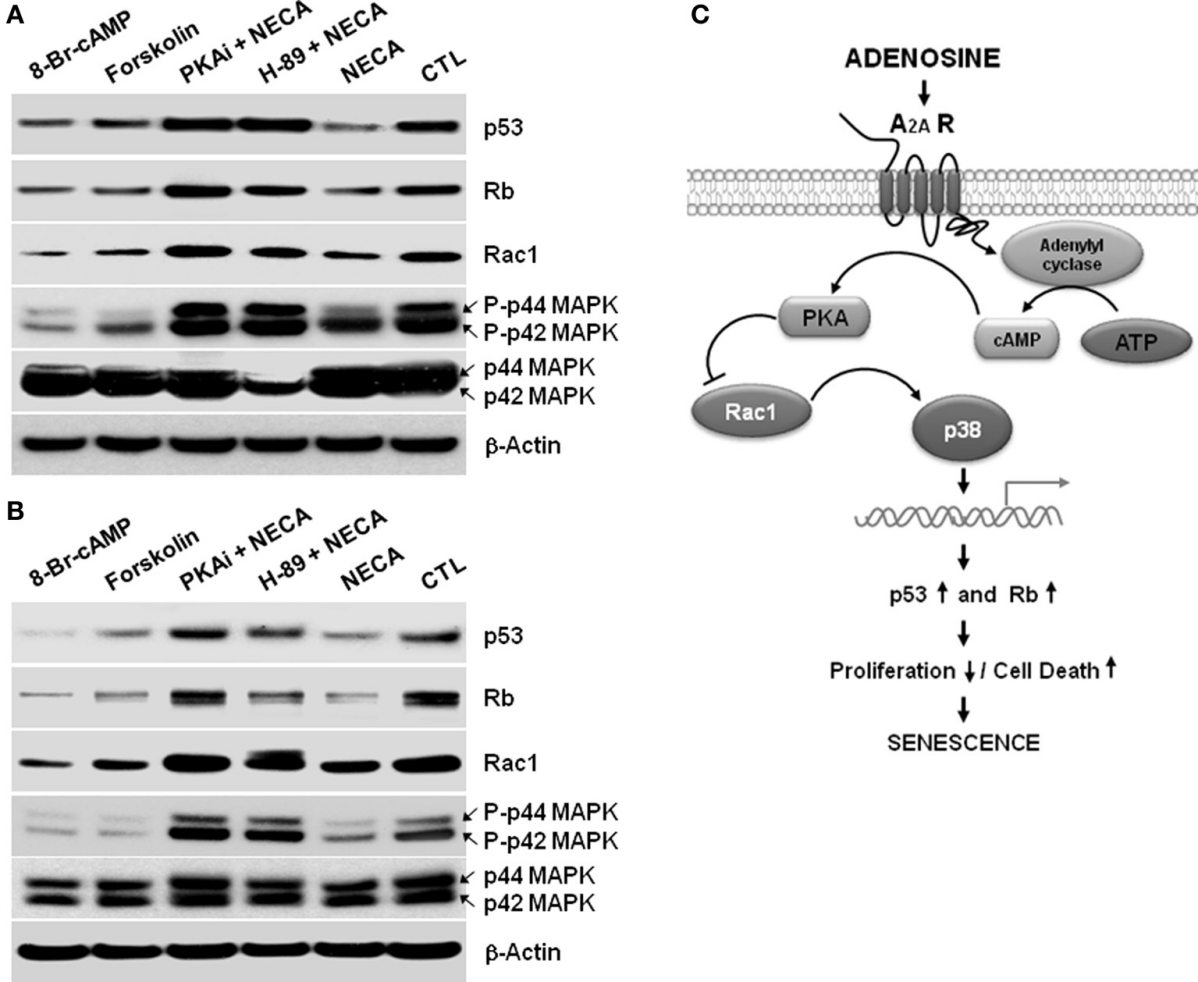
**A cAMP analog, or an activator of adenylyl cyclase Forskolin, or NECA down regulates p53 and Rb through down regulation of Rac1 and decreased activation of p38 MAPK in the HSC**. LX-2 cells were treated for 2 days, and 10 days cultured activated primary rat HSC were treated for 5 days with 8-Bromo-cAMP (1mM) or, Forskolin (50 μM) or NECA (10 μM) in presence or absence of PKA inhibitor 12–14 amide (myristoylated; 10 μM) or H-89 dihydrochloride hydrate (25 μM). **(A)** Western blot analysis of p53, Rb, Rac1, and p44/42 MAPK levels in the 2 days treated LX-2 cells. **(B)** Western blot analysis of p53, Rb, Rac1, and p44/42 MAPK levels in the 5 days treated activated rat primary HSC. **(C)** Summary of adenosine receptor A_2A_ mediated signaling in the process of senescence in HSC.

We next wished to identify the pathway between A_2A_ receptor and the p53 and Rb signaling cascades to understand how NECA mediates its regulation of HSC. In other systems NECA exerts its effect via Gα_s_-dependent activation of adenylyl cyclase resulting in an increased cytosolic cAMP followed by activation of PKA (Grant et al., 1999, 2001). We used 8-Bromo-cAMP, an analog cAMP which is a known activator of PKA, and an adenylyl cyclase activator forskolin to test whether by activation of adenylyl cyclase, cAMP and PKA can reproduced the downregulation of p53 and Rb, which is showed by the treatment of NECA. Treatment with 8-Bromo-cAMP (1 mM) and forskolin (50 μM) both down regulated the expression of p53 and Rb in LX-2 (Figure 4A) and primary rat HSC (Figure 4B). This demonstrates that the known adenylyl cyclase/cAMP/PKA pathway downstream of A_2A_ receptor is able to induce downregulation of p53 and Rb. We further confirmed this pathway by using pharmacologically available PKA inhibitors PKi 14–22 amide (10 μM) or H-89 dihydrocloride hydrate (25 μM). Both PKA inhibitors blocked the suppression of p53 and Rb by NECA (Figure 4). It is also known that the activation of p38 MAPK results in accumulation of p53 and Rb protein level, resulting in senescence (Wang et al., 2002). We checked whether the NECA, 8-Bromo-cAMP, or forskolin-mediated down regulation of p53 and Rb were from deactivation of p38 MAPK and/or down regulation of upstream of p38 MAPK, Rac1. Figure 4 shows that NECA, 8-Bromo-cAMP, or forskolin down regulated the protein level of Rac1 and phosphorylation of p44/42 MAPK. This downregulation by NECA was blocked by additional treatment of the PKA inhibitor PKAi or H-89. These data show that activation of AR-A_2A_ by NECA down regulates the p53 and Rb level through PKA/Rac1/p38 MAPK pathway (Figure 4C).

## Discussion

Adenosine is recognized as having an important role in HSC differentiation and liver fibrosis (Cronstein, 2004; Chan et al., 2006; Sohail et al., 2009; Chan and Cronstein, 2010). In response to adenosine, HSC up-regulate collagen and transforming growth factor, and inhibition of the adenosine A_2A_ receptor signaling decreases liver fibrosis *in vivo* (Chan et al., 2006; Chunn et al., 2006; Che et al., 2007). We have also recently demonstrated that adenosine inhibits platelet-derived growth factor–mediated HSC chemotaxis (Hashmi et al., 2007). The rapid production of adenosine in sites of tissue injury and hypoxia, coupled with its rapid removal by ectonucleotidases makes adenosine an excellent messenger to link injury with tissue repair responses. Because of their anti-fibrotic potential several adenosine receptor antagonists are in clinical development (Gao and Jacobson, 2007).

After activation HSC undergo rapid proliferation followed by apoptosis or senescence, and each of these cell fates is important at the level of the whole organ (Krizhanovsky et al., 2008b; Friedman, 2010). For example inhibition of the senescence pathway by deleting p53 results in fewer senescent HSC, a greater number of HSC, increased liver fibrosis and slower resolution of fibrosis (Krizhanovsky et al., 2008b). The *in vivo* situation is complex because these are simultaneous rather than sequential events. With the recognition of the important role of HSC proliferation, apoptosis and senescence in liver fibrosis it becomes important to ask how these steps are regulated. To address this we examined the role of adenosine in regulating these different HSC fates. We initially chose to focus on p53 and p16/Rb tumor suppressor pathways. The reason for this is that both pathways are central to the development of senescence and their regulation would give an early indication if adenosine has a role in senescence biology (Campisi and d'Adda di Fagagna, 2007; Collado et al., 2007). P53 promotes senescence by transactivation of a number of genes that inhibit proliferation (He et al., 2007). P16 inhibits cyclin-dependent kinases 2 and 4, preventing Rb phosphorylation and allowing Rb to promote a heterochromatin environment that silences proliferation associated genes (Narita et al., 2003). The pan-adenoisne agonist NECA significantly suppressed both p53 and Rb in the LX-2 cell line as well as primary rat HSC. The known requirement for the adenosine A_2A_ receptor in liver fibrosis made it the prime candidate, and was demonstrated to be responsible for p53 and Rb suppression.

Downregulation of p53 and Rb predicted that adenosine signaling will significantly increase proliferation and reduce apoptosis. This was confirmed by quantifying BrdU incorporation and annexin/PI staining for LX-2 and primary rat HSC. The specificity of the A_2A_ receptor for proliferation and cell death was again demonstrated by the use of receptor specific agonists and antagonists. A previous study has reported that adenosine signaling does not affect HSC proliferation, however this was limited to examining cell lines and lower concentrations of agonists and antagonists (Che et al., 2007). Our results demonstrate that adenosine downregulated core cell fate pathways of p53 and Rb, increased HSC proliferation, and reduced HSC apoptosis. The increase in HSC proliferation, even with a block in cell death, would not be expected to result in greater HSC number and liver fibrosis because proliferating cells enter senescence after a variable number of cell cycles (Hoare et al., 2010). This was demonstrated to be the case as the majority of primary rat HSC cultured for 21 days entered senescence, and this was associated with an increase in p53 and Rb. In contrast primary rat HSC cultured for 21 days in the presence of NECA demonstrated lower levels of p53 and Rb and significantly less senescence.

The above data set establish an important role for adenosine in regulating the commitment of a HSC population between proliferation, apoptosis and senescence. Each of these processes has been shown to be important in HSC biology and liver fibrosis, and we propose that by shifting the balance between them adenosine has an amplified effect on the overall HSC population and liver fibrosis. In cells other than HSC IL-6 and IL-8 have been shown to induce senescence via STAT5 and suppressor of cytokine signaling (Kuilman et al., 2008). In HSC interleukin-22 has been demonstrated to inhibit HSC activation and apoptosis but to induce HSC senescence via a STAT3 and SOCS3 mediated pathway (Kong et al., 2012). Thus IL-22 would limit the numbers of HSC entering the activated pool, and would also accelerate their removal by increasing senescence. Adenosine by contrast increases the entry of HSC into the activated pool, and keeps them there by decreasing cell death and senescence.

Adenosine signals via four receptors, A_1_, A_2A_, A_2B_, and A_3_. Of these A_2A_ is required for the development of liver fibrosis, which fits very well with our demonstration of a role for A_2A_ receptors in the adenosine mediated effects on HSC fate decisions. A stimulatory cAMP coupled pathway is known to be present downstream of A_2A_ receptor. By using a number of stimulators and inhibitors we have demonstrated that an adenyly cyclase, cAMP, PKA, Rac1, and p38 pathway is responsible for the downregulation of p53 and Rb. A number of adenosine antagonists are under clinical development as anti-fibrotics and the identification of this pathway makes available additional targets therapeutic intervention.

In summary we have identified adenosine as the first HSC anti-senescence factor. In addition adenosine regulates HSC fate decisions of proliferation and cell death. When coupled with the known ability of adenosine-A_2A_ receptor to induce HSC activation and collagen production it explains the pro-fibrotic qualities of this pathway, and supports prioritizing it as a target for therapeutic development.

### Conflict of interest statement

The authors declare that the research was conducted in the absence of any commercial or financial relationships that could be construed as a potential conflict of interest.

## Supplementary material

Supplemental Figure 1 **NECA enhances cellular longevity in the primary rat HSC**. Cells were cultured without (CTL/DMSO) or with 10 μM of NECA for 60 days. **(A)** Rat primary HSC were cultured for 60 days without (CTL) or with NECA. **(B)** The number of viable cells was quantitated using Image-J cell counts in randomly taken images of 60 days cultured rat primary HSC without (CTL) or with NECA (10 μM) using inverted phase contrast microscope.

Supplemental Figure 2 **NECA suppresses long-term culture-induced β-Gal expression in the LX-2 cells**. Arrows are indicating expression of β-Gal in the nucleus of LX-2. **(A)** β-Gal expression in 2–40 passages normal culture of LX-2 cells. **(B)** β-Gal expression in the 40 passages culture of LX-2 cells treated without (CTL) or with 10 μM of NECA.

## Abbreviations

AR-A_1_: adenosine receptor A_1_
AR-A_2A_: adenosine receptor A_2A_
AR-A_2B_: adenosine receptor A_2B_
AR-A_3_: adenosine receptor A_3_
β-gal: beta-galactosidase
cAMP: cyclic adenosine monophosphate
HSC: hepatic stellate cells
NECA: 5′-N-Ethylcarboxamide adenosine
PKA: protein kinase A
SA-β-Gal: senescence associated β-galactosidase.
