# Supplementary figures and images for: Activation of adenosine receptor A2A increases HSC proliferation and inhibits death and senescence by down-regulation of p53 and Rb
Source: Front Pharmacol. 2014 Apr 10;5:69. doi: 10.3389/fphar.2014.00069 (PMC3989592; doi:10.3389/fphar.2014.00069)

## Supplemental Fig.1

**A**

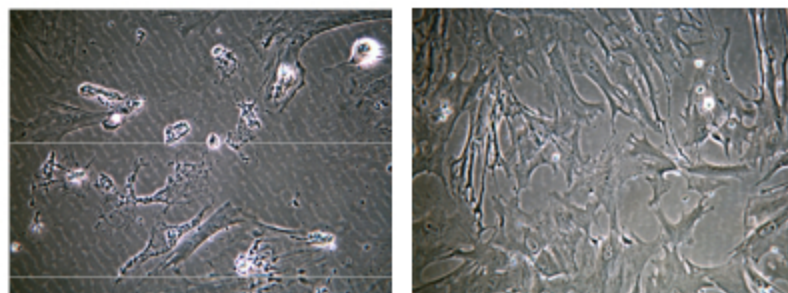

**CTL**

**NECA**

**B**

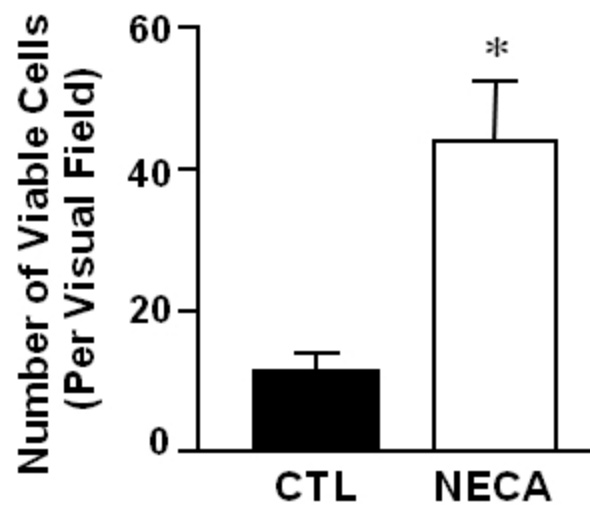

Supplement: Supplemental Figure 1 — NECA enhances cellular longevity in the primary rat HSC. Cells were cultured without (CTL/DMSO) or with 10 μM of NECA for 60 days. (A) Rat primary HSC were cultured for 60 days without (CTL) or with NECA. (B) The number of viable cells was quantitated using Image-J cell counts in randomly taken images of 60 days cultured rat primary HSC without (CTL) or with NECA (10 μM) using inverted phase contrast microscope. [file Presentation1.PDF]

## Supplemental Fig.2

**A**

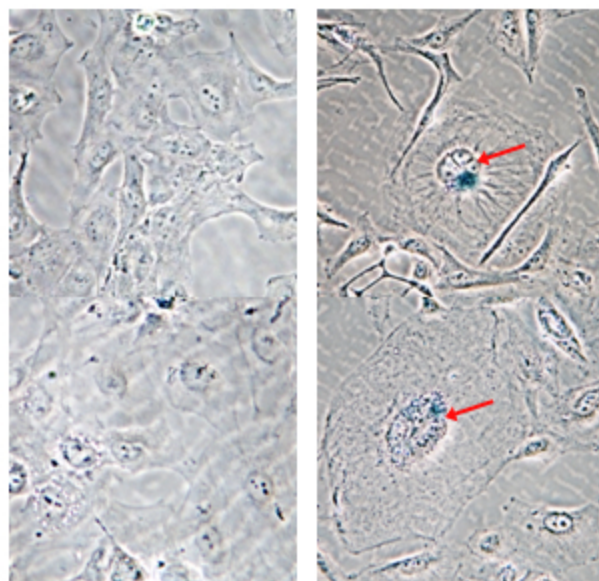

Passages 2

40

**B**

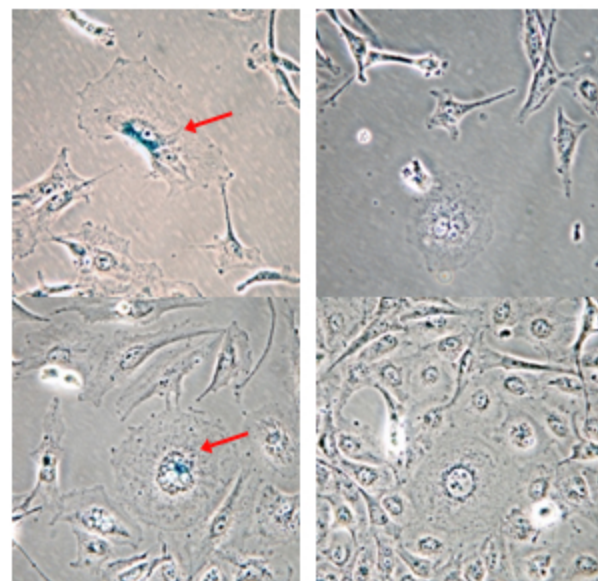

Passages 40/CTL

40/NECA

Supplement: Supplemental Figure 2 — NECA suppresses long-term culture-induced β-Gal expression in the LX-2 cells. Arrows are indicating expression of β-Gal in the nucleus of LX-2. (A) β-Gal expression in 2–40 passages normal culture of LX-2 cells. (B) β-Gal expression in the 40 passages culture of LX-2 cells treated without (CTL) or with 10 μM of NECA. [file Presentation2.PDF]
